# Anterior cruciate ligament—Return to sport after injury scale brief version after ACL reconstruction: Persian translation, cross‐cultural adaptation and validation

**DOI:** 10.1002/jeo2.12074

**Published:** 2024-07-08

**Authors:** Nasim Eshraghi, Peyman Mirghaderi, Reza Omid, Mohamad Sajadi, Amirreza Pashapour‐Yeganeh, Reza Hosseini‐Dolama, Payman Rahimzadeh, Alireza Moharrami, Amir Rakhshan, Seyed Mohammad Javad Mortazavi

**Affiliations:** ^1^ Surgical Research Society (SRS), Students' Scientific Research Center Tehran University of Medical Sciences Tehran Iran; ^2^ Joint Reconstruction Research Center Tehran University of Medical Sciences Tehran Iran; ^3^ Vali‐E‐Asr Reproductive Health Research Center, Family Health Research Institute Tehran University of Medical Sciences Tehran Iran; ^4^ Department of Foreign Languages Tehran University of Medical Sciences Tehran Iran

**Keywords:** AC‐RSI, anterior cruciate ligament, Persian, questionnaire, reliability, validity

## Abstract

**Purpose:**

The purpose of this study is to analyze the short anterior cruciate ligament return to sport after injury (ACL‐RSI) (Persian) version's cultural adaption and validity.

**Methods:**

To assess test–retest reliability, 102 participants were filled out the short ACL‐RSI(Per) scale 6 months or more after ACLR surgery. Internal consistency (Cronbach's alpha), test–retest reliability (intraclass correlation coefficients), construct validity (Pearson's *r*) and sensitivity (floor/ceiling effect) were determined. In addition, patient completed other relevant measures such as Lysholm scores, the hospital for special surgery ACL satisfaction survey (HSS ACL‐SS), the visual analogue scale (VAS) of pain and patient's satisfaction, the Tegner activity score (TAS), the single assessment numeric evaluation (SANE) and the Cincinnati Knee Rating System (CKRS).

**Results:**

The short ACL‐RSI(Per) scale showed high internal consistency (Cronbach's alpha = 0.91) and test–retest reliability (ICC = 0.923). Significant correlations between short ACL‐RSI(Per) and other scales supported validity. There was a statistically significant connection between the short ACL‐RSI(Per) and the following outcomes: HSS ACL‐SS (*r* = 0.698, *p* < 0.001), VAS pain (*r* = 0.356, *p* < 0.001), CKRS (*r* = 0.644, *p* < 0.001), TAS (*r* = 0414, *p* < 0.001), Lysholm score (*r* = 0.467, *p* < 0.001) and SANE score (*r* = 0.536; *p* < 0.001). In addition to a satisfactory ceiling impact (15%), a sizeable floor effect (16.7%) was also seen.

**Conclusion:**

The short ACL‐RSI(Per) scale is a reliable and valid tool for assessing psychological readiness for return to sport after ACL reconstruction in Persian.

**Level of Evidence:**

III.

**Abbreviations**
ACLR: anterior cruciate ligament reconstruction
ACL‐RSI: anterior cruciate ligament return to sport after injury
CKRS: Cincinnati Knee Rating System
HSS ACL‐SS: hospital for special surgery ACL satisfaction score
ICC: intraclass correlation coefficient
PROM: patient‐reported outcome measure
SANE: single assessment numeric evaluation
TAS: Tegner activity scale
VAS: visual analogue scale

## INTRODUCTION

Individuals with elevated susceptibility to knee instability, who express a desire to resume athletic activities, generally undergo a surgical procedure known as anterior cruciate ligament reconstruction (ACLR) [22, 30]. Based on a recent study, around 80% of athletes who receive ACLR surgery can resume participating in sports. However, their outcomes are not significantly better than those who receive nonoperative treatment [28]. Nevertheless, a significant number of patients necessitate the adjustment of their physical activities, opting for less strenuous exercise, engaging in sports that do not include sudden changes in direction or sharp turns and/or refraining from the specific activity that led to the injury [28]. An analysis of multiple research studies revealed that the rate of returning to preinjury levels ranged from 63% to 95%. It was noticed that female athletes and those with shorter follow‐up periods had lower rates of return. In addition, the study found that the average duration from surgery to receiving clearance to return to sport (RTS) was 10.6 ± 4.4 months [10].

Various factors influence the ability of patients with a damaged ACL to RTS. Several factors, including reinjury anxiety, psychological readiness and high rehabilitation desire, have an impact on the RTS process [36, 45]. Additional criteria encompass the severity of the injury, the patient's age, gender and level of physical activity, the specific type of graft employed, and the rehabilitation regimen [28]. Furthermore, the ability to resume athletic activities following ACL repair surgery can be significantly impacted by psychosocial elements, such as one's expectations and beliefs regarding the rehabilitation process, anxiety related to performance and inherent personal traits [7]. Psychological readiness is a crucial determinant for achieving favourable results in patients resuming sports activities following ACL damage. Multiple studies have emphasized the significance of psychological readiness in resuming athletic activities following ACL injury and reconstruction. Studies have demonstrated that a higher level of psychological readiness is linked to the resumption of sports activities in both teenage and adult athletes following ACLR surgery [11, 44]. Psychological readiness for RTS can be influenced by various factors, including fear of reinjury, lack of self‐confidence, kinesiophobia, sadness and anxiety [33]. Moreover, the ACL‐RSI scale has been employed to evaluate psychological reactions, specifically the level of confidence in the injured body part and the assessment of risk. This scale has proven to be a valuable instrument in determining patients’ readiness to resume sports activities following an ACL injury [33]. Hence, it is crucial to consider psychological readiness as a significant component of comprehensive rehabilitation and reintegration into sports for individuals who have suffered ACL damage.

The ACL Return to Sport after Injury (ACL‐RSI) scale, developed in 2008, is a widely used instrument for evaluating psychological readiness for returning to sport after ACLR [26]. The original 12‐item scale has been translated and validated in various languages [1, 5, 13, 17, 24, 25, 27, 31, 35]. The shorter 6‐item version has also been developed, demonstrating comparable reliability, predictive ability and responsiveness [42, 43]. In busy clinical settings, it can be very beneficial to utilize this tool to identify athletes who may encounter difficulties while attempting to resume their sport [42]. The short version demonstrates strong internal consistency, and despite being half the length of the original scale, it maintains its reliability, predictive ability and responsiveness [42]. The aim of this study was to shorten the ACL‐RSI and assess its divergent and predictive validity in comparison to the original and lengthier forms to ascertain its potential utility for the Iranian population.

## METHODS

### Study design

The subjects of a cross‐sectional study were patients who had undergone ACLR surgery at our tertiary referral centre (Imam Khomeini Hospital Complex) from 2018 to 2022. The inclusion criteria were as follows:

1. Individuals who underwent primary ACLR surgery, with or without meniscal injury, for an ACL rupture of any aetiology, regardless of whether it was sports‐related or not.
2. Individuals who had undergone surgery at least 6 months prior.
3. Individuals who were fluent in all three components of the Persian language.

The study excluded patients who had previously undergone articular cartilage repair, experienced bilateral ACL injuries or undergone multifilament restoration. The short ACL‐RSI(Per) scale and other measures were administered to patients at their most recent postoperative follow‐up visit, which was a minimum of 6 months following surgery.

### Translation and cross‐cultural adaptation

**Figure 1:**
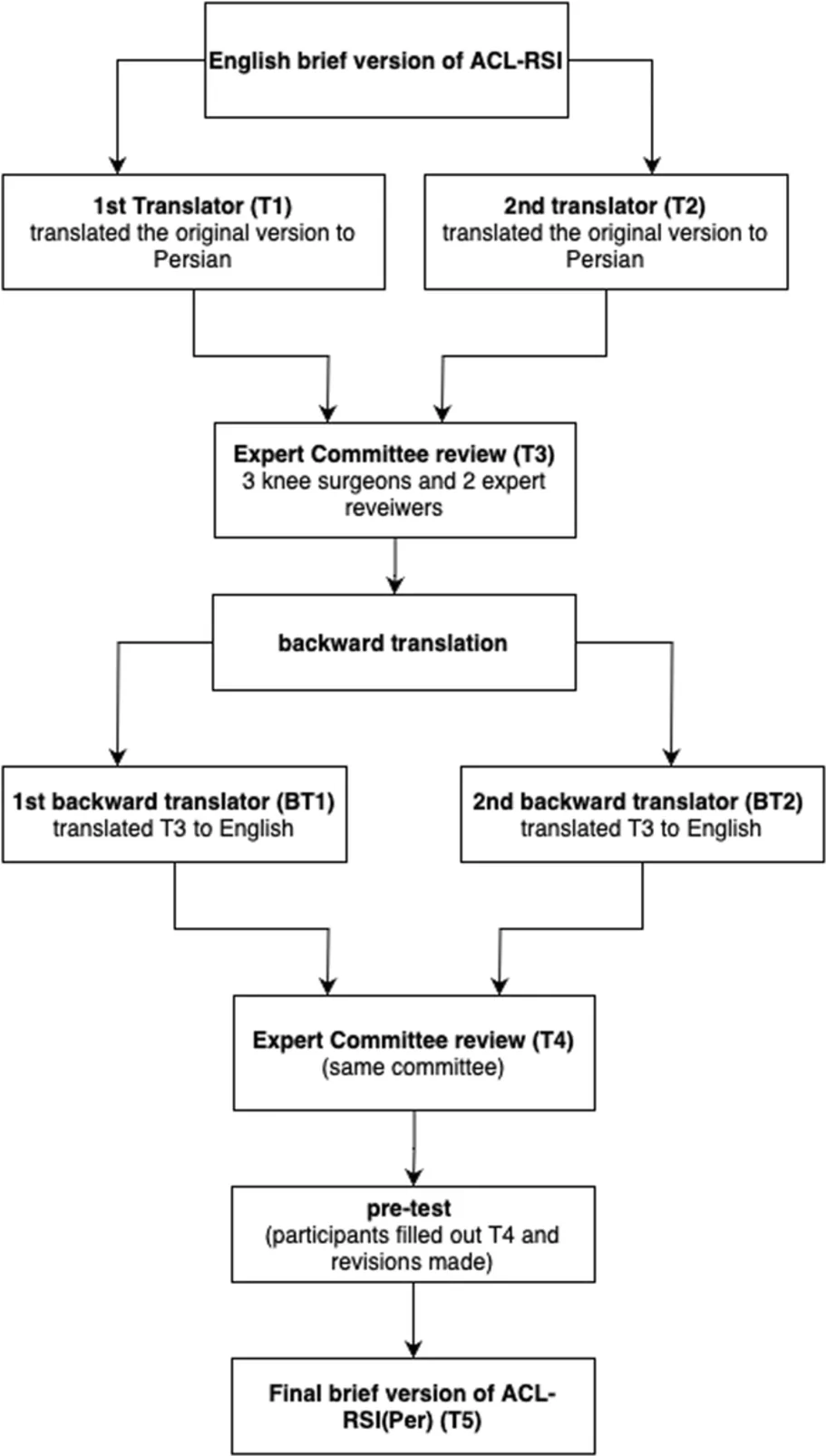
Flow diagram of the study process and enrollment of participants.

Guillemin et al. developed a framework for modifying scoring systems to be applicable in different languages and cultures [12]. The letter followed all of the preceding directions. The research comprises two components: the collection of data from patients to assess reliability and cross‐cultural adaptability and the translation of the questionnaire into Persian and then back into English. The initial step consisted of forward translation, consensus and correction, as well as reverse translation, committee evaluation and preliminary testing (Figure 1) [20].

The short ACL‐RSI was translated into Persian by two different translators, T1 and T2. T1 possessed medical expertise and however T2 lacked it. The initial translations underwent scrutiny by knee surgeons and experts. T2 exhibited a resemblance to T1. A mutually accepted Persian version (T3) was formulated. Two blind translators (BT1 and BT2) performed reverse translations into English. The expert committee evaluated the original scale by comparing it to the forward (T1, T2) and backward translations (BT1, BT2). The final version of the Persian short ACL‐RSI (T4) was developed based on Figure 1.

### Pretest

Prior to conducting the survey, 10 patients were provided with the definitive version of the questionnaire and were closely monitored as they completed it. Subsequently, all participants were surveyed regarding the questionnaire's clarity and readability. The comprehension of the question was assessed on a scale ranging from 0 (indicating a complete lack of understanding) to 2 (indicating a thorough understanding). The patients’ mean scores were 12, demonstrating their adequate proficiency in solving the study's problems. We took note of their suggestions for enhancing the design and usability of the scale. T4 underwent modifications and enhancements in response to the feedback provided by the test subjects. T5 represents the ultimate iteration that has been compiled and is currently accessible for execution (Supporting Information S2 file).

### Patients and scores

In order to adequately assess the construct validity, repeatability, ceiling and floor effects and internal consistency, it was necessary to have a sample size of at least 100 patients, as recommended by Terwee et al. [23, 38]. The analysis comprised a total of 102 participants who were requested to complete pain and satisfaction evaluations, including the single assessment numeric evaluation (SANE) and the visual analogue scale (VAS), along with the validated editions of the Lysholm, Cincinnati Knee Rating System (CKRS) [21], Tegner activity scale (TAS) and visual analogue scale (VAS).

Webster et al. [42]. effectively developed a concise 6‐item version of the ACL‐RSI, comprising three domains and their respective questions. The short version comprises three elements for the ‘Emotion’ subscale, two for the ‘Confidence in performance’ subscale and one for the ‘Risk appraisal’ subscale. These factors impact a patient's psychological readiness to resume sporting activities after undergoing ACLR surgery. Only the short version was administered, without a direct comparison to the original version.

The HSS ACL‐SS, a satisfaction scale based on patient feedback [15, 19], consists of 10 variables that assess the level of happiness experienced by patients following their ACLR surgery. Subjects provide a numerical value to each query on a scale of 1–5. The Lysholm questionnaire utilizes a scoring scale that spans from 0 to 100 and comprises a total of 8 inquiries [18, 37]. The question ‘How would you rate your injured body part on a scale of 0 to 100?’ constitutes a single numeric assessment (SANE), a subjective rating scale used to evaluate patients' results [46, 47]. The International Knee Documentation Committee (IKDC) score and the Knee Outcome Survey (KOS) have been found to have a strong correlation with the SANE score [6, 34]. The VAS is a dependable and uniform approach for quantifying a patient's pain intensity on a continuum ranging from 0 to 10 [4].

### Ethical considerations

This project has received authorization from our school's Institutional Review Board (IRB) for health research. (Obfuscated) Authorization ID. Patients gave their informed verbal permission after being told of the study's goals. We certify that a data availability statement has been included in the main paper file, following the templates and examples provided in the instructions for authors.

### Statistical analysis

The statistical analyses were conducted using SPSS version 23. Mean and standard deviation were used to represent continuous values, whereas frequencies were used to report categorical variables. Cronbach's alpha was used to evaluate internal consistency [38]. The assessment of test–retest reliability was conducted by calculating intraclass correlation coefficients (ICCs). The construct validity was assessed by calculating Spearman's rank correlation coefficients between the ACL‐RSI scores and scores on the HSS ACL‐SS, Lysholm score, CKRS, TAS, SANE and VAS. Correlation coefficients ranging from 0.3 to 0.5 were classified as moderate, while correlations greater than 0.5 were considered strong [9]. To confirm content validity, the presence of floor and ceiling effects was assessed. It was judged acceptable if less than 15% of patients scored either the minimum or maximum values [2].

## RESULTS

### Patient characteristics

The analysis comprised a total of 102 patients who underwent ACLR. The sample consisted of 90 men and 12 women, with a mean age of 29.1 ± 8.0 years. Out of the individuals who experienced an ACL tear, 54% had engaged in high‐risk sports such as basketball, soccer, football, lacrosse, skiing or volleyball prior to the injury. Further details regarding the participants' demographics and clinical characteristics can be accessed at this location (Table 1).

**Table 1:** Characteristics of the study population.

Variable (*N* = 102)	Mean ± SD or *n* (%)
Age, years	29.1 ± 8.0
Sex	
Male	90 (88.2)
Female	12 (11.8)
Follow‐up	2.5 ± 1.2 (range: 1–6)
Side	
Left	40 (39.2)
Right	62 (60.8)
Charlson Comorbidity Index	0.1 ± 0.30
BMI, kg/m^2^	22.7 ± 3.4
Mechanism	
Sport	65 (63.7)
Contact	16 (15.7)
Noncontact	49 (48.0)
Accident	21 (20.6)
Falling	12 (11.8)
Chronic	4 (3.9)
Sports before Injury	
Soccer	54 (52.9)
Other	17 (16.7)
None	31 (30.4)
High‐risk sports for ACL tear (pivoting sports)	
Yes	56 (54.9)
No	15 (14.7)
None	31 (30.4)
Preinjury level of sport	
Competitive	24 (23.5)
Recreational	47 (46.1)
None	31 (30.4)
Time from injury to surgery (months)	21.9 ± 39.8 (range: 0.2–240)
Postoperative scores	
ACL‐RSI	30.5 ± 27.1
Emotion	30.1 ± 31.3
Confidence in performance	30.5 ± 31.3
Risk appraisal	31.5 ± 30.8
Tegner	Median = 5 (range: 0–10)
Lysholm	73.5 ± 19.3
VAS pain	2.2 ± 2.7
Cincinnati	65.6 ± 22.7
SANE	60.2 ± 24.1
HSS ACL‐SS	26.9 ± 11.3

Abbreviations: ACL‐RSI, anterior cruciate ligament return to sport after injury; BMI, body mass index; HSS ACL‐SS, hospital for special surgery ACL satisfaction; SANE, single assessment numeric evaluation; SD, standard deviation; VAS, visual analogue scale.

### Reliability

The Cronbach's alpha coefficient for the overall score of the ACL‐RSI was 0.901. The performance subscale had a high level of internal consistency, with a coefficient of 0.800, based on two items. Similarly, the emotion subscale demonstrated a strong internal consistency, with a coefficient of 0.908 (Table 2). The results of Cronbach's alpha are shown by systematically removing each item one at a time.

**Table 2:** Item‐total statistics for each item of the questionnaire.

Item	Scale mean if item deleted	Scale variance if item deleted	Corrected item‐total correlation	Cronbach's alpha if item deleted
ACL‐RSI 1	14.70	187.639	0.686	0.903
ACL‐RSI 2	15.13	195.657	0.685	0.902
ACL‐RSI 3	15.17	180.437	0.820	0.883
ACL‐RSI 4	15.75	197.910	0.743	0.896
ACL‐RSI 5	15.02	183.445	0.736	0.896
ACL‐RSI 6	15.61	175.369	0.838	0.880

Abbreviation: ACL‐RSI, anterior cruciate ligament return to sport after injury.

The aggregate ACL‐RSI score had a high test–retest ICC of 0.923 (*p* = 0.001). The ICC for the emotion subscale was 0.926, indicating its highest level of significance. Conversely, the ICC for the risk appraisal subscale was 0.880, indicating its lowest level of significance. The information is shown in Table 3. All ICCs exceeded the threshold of 0.80, indicating a significantly high level of agreement. Consequently, the Persian short ACL‐RSI test maintained its accuracy.

**Table 3:** Spearmen correlation coefficients between the test (t) and retest (rt) of the ACL‐RSI scale to assess the test‐retest reliability.

ACL‐RSI subscales	Emotion (rt)	Confidence in performance (rt)	Risk appraisal (rt)	Total (rt)
Emotion (t)				
*r*	0.926	‐	‐	‐
*p* Value	<0.001			
Confidence in performance (t)				
*r*	‐	0.911	‐	‐
*p* Value		<0.001		
Risk appraisal (t)				
*r*	‐	‐	0.880	‐
*p* Value			<0.001	
Total (t)				
*r*	‐	‐	‐	0.923
*p* Value				<0.001

Abbreviation: ACL‐RSI, anterior cruciate ligament return to sport after injury.

### Validity

The floor and ceiling impact, which indicates content validity, was calculated by determining the percentage of patients who scored either the minimum (0) or maximum (100) on each item. Floor effect: Zero was the score for seventeen patients, which accounted for 16.7% of the total. One case, however, achieved a flawless score of 100, indicating a ceiling effect, which accounted for 1.0% of the total. Consequently, the short ACL‐RSI(Per) was impacted by a floor effect, exceeding 15%.

**Figure 2:**
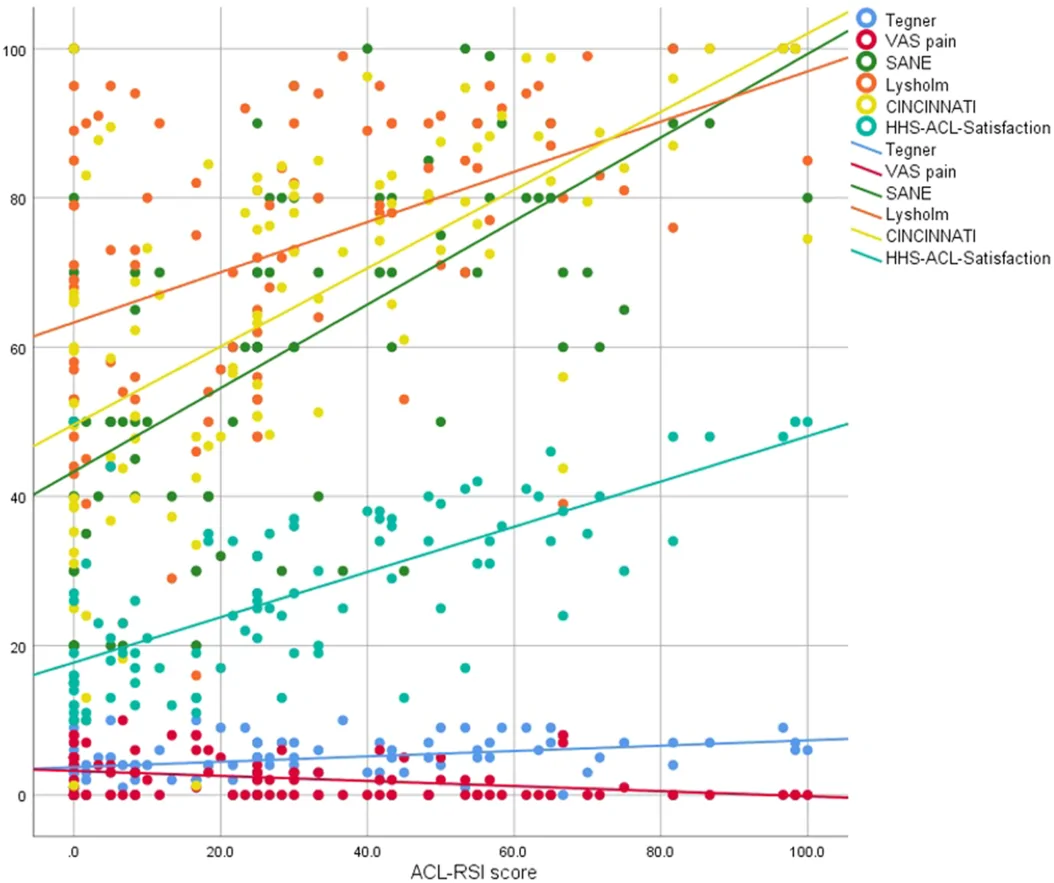
Significant strong/moderate correlation between the total short ACL‐RSI(Per) score and other relevant scores. ACL‐RSI, anterior cruciate ligament return to sport after injury; HSS ACL‐SS, hospital for special surgery ACL satisfaction; SANE, single assessment numeric evaluation; VAS: visual analogue scale.

The Lysholm (*r* = 0.467), CKRS (*r* = 0.644), TAS (*r* = 0.414), SANE (*r* = 0.536), HSS ACL‐SS (*r* = 0.698) and VAS pain ratings (*r* = −0.356) showed significant correlations (Strong or moderate) with the total short ACL‐RSI(Per) score (all *p* < 0.001) (Figure 2). By examining the correlation coefficients of HSS ACL‐SS, CKRS and ACL‐RSI domains, we may observe a more pronounced association between the two very similar portions. Question 2 of the HSS ACL‐SS, which measures confidence in one's knee for athletic activities, showed a strong positive correlation with the ACL‐RSI confidence in performance score (*r* = 0.681, *p* < 0.001). Similarly, questions 6 and 7, which assess returning to the same level of sport and previous level of competition, also exhibited a significant correlation (Table 4). The correlation between jumping and twisting and the total ACL‐RSI score was stronger (*r* = 0.567, *r* = 0.566, *p* < 0.001) compared to other CKRS domains (*r* = 0.510).

**Table 4:** Spearmen correlation coefficients between different domains of the ACL‐RSI and relevant functional scores.

ACL‐RSI subscales	Emotion	Confidence in performance	Risk appraisal	Total
Lysholm				
*r*	0.436	0.461	0.308	0.467
*p* Value	<0.001	<0.001	<0.001	<0.001
Cincinnati				
r	0.608	0.650	0.421	0.644
*p* Value	<0.001	<0.001	<0.001	<0.001
Tegner				
*r*	0.344	0.423	0.405	0.414
*p* Value	<0.001	<0.001	<0.001	<0.001
SANE				
*r*	0.552	0.482	0.392	0.536
*p* Value	<0.001	<0.001	<0.001	<0.001
HSS ACL‐SS				
r	0.646	0.725	0.530	0.698
*p* Value	<0.001	<0.001	<0.001	<0.001
VAS pain				
*r*	−0.361	−0.397	−0.171	−0.356
*p* Value	<0.001	<0.001	0.09	<0.001

Abbreviations: ACL‐RSI, anterior cruciate ligament return to sport after injury; SANE, single assessment numeric evaluation; VAS, visual analogue scale.

## DISCUSSION

The primary outcome of this study was the validation of the Persian translation of the short ACL‐RSI questionnaire as a feasible and dependable tool for assessing patients who have undergone ACLR. The overall ACL‐RSI score in our study demonstrated a strong test–retest reliability, as indicated by an ICC value of 0.923. The mood subscale had the highest level of dependability, with an ICC of 0.926. The ICC values for all measurements exceeded 0.80, indicating a high level of reliability. The results of this study show that the ICCs reported for the Persian [27], French [5], Spanish [31], Dutch [25], Portuguese [35], Arabic [1], Swedish [17] and Turkish [13] versions of the ACL‐RSI range from 0.78 to 0.99, which is comparable to other language variants. The obtained ICC is in close proximity to the ICCs of 0.93 and 0.916 for the Dutch and Japanese translations of the ACL‐RSI, respectively (Table 5).

**Table 5:** Test–retest reliability, internal consistency and validity of the ACI‐RSI in different languages.

Study and year	Language	Population	Test–retest reliability (ICC)	Cronbach's alpha	Validation scores	Correlation (Pearson's *r*)
Alzhrani (2022) (Short version)	Arabic	60	0.871	0.734	IKDC	0.515
					KOOS	0.247–0.590
Albano (2022) (Short version)	Portuguese	168	0.85	0.78	IKDC	0.52
					TSK‐17	−0.45
Pirayeh (2023)	Persian	100	0.90	0.94	IPRRS	0.76
					TSK‐17	−0.68
					IKDC	0.44
Silva (2017)		100	0.78	0.87	IKDC	−0.58
					TSK	−0.51
Jia (2018)	Chinese	122	0.98	0.94	IKDC	0.46
					KOOS	−0.30
Chen (2017)	Simplified Chinese	112	0,90	0.96	Tegner	0.695
					KOOS	0.66
					TSK	−0.678
					IKDC	0.463
Slagers (2016)	Dutch	150	0.93	0.94	IPRRS	0.79
					IKDC	0.51
					TSK	−0.46
					MHLC‐C	0.72
					KOOS	0.48
Bohu (2013)	French	91	0.90	0.96	IKDC	0.42
					KOOS	0.22
Tortoli (2020)	Italian	129	0.99	0.94	IKDC	0.34
					TSK	−0.42
					SF‐12	−0.40
					MCS12	0.40
					PCS12	0.44
Hirohota (2020)	Japanese	93	0.916	0.912	IKDC‐SKF	0.41
					Lysholm	0.21
					KOOS	0.26
					TSK	−0.34
Ku Ha (2018)	Korean	129	0.949	0.932	IKDC‐SFK	0.573
					KOOS	0.679
					Lysholm	0.535
Salakite (2019)	Lithuanian	65	‐	0.94	IKDC	0.637
					Tegner	0.303
Faleide (2020)	Norwegian	197	0.94	0.95	IKDC	0.61
					TSK	0.34
					KOOS	0.66
Sala‐Barat (2019)	Spanish	114	0.9	0.9	KOOS	0.6
					TSK	0.5
					I‐PRRS	0.8
Kvist (2011)	Swedish	82	0.893	0.948	KOOS	0.718
					TSK	0.689
					KSES	0.705
Harput (2016)	Turkish	93	0.92	0.86	I‐HLC‐C	0.287
					IKDC	0.44
					TSK	−0.45
					KOOS	0.58
					Lysholm	r=0.45

Abbreviations: I‐PRRS, Injury‐Psychological Readiness to Return‐To‐Sport scale; IKDC, International Knee Documentation Committee Score; IKDC‐SKF international knee documentary committee subjective knee form; KOOS, Knee injury and Osteoarthritis Outcome Score; MCS12 mental component summary score; MHLC‐C, Multidimensional Health Locus of Control, C form; PCS12 physical component summary score; TSK, Tampa Scale of Kinesiophobia.

The results of our study revealed strong associations between the overall Persian ACL‐RSI score and several other outcome measures, such as Lysholm, CKRS, TAS, SANE, HSS ACL‐SS and VAS pain ratings. These associations provide evidence for the scale's construct validity. The correlation coefficient between Lysholm and the Turkish (*r* = 0.45) and Korean (*r* = 0.535) versions closely aligns with the stated values, suggesting strong cross‐cultural validity. This information may be found in Table 5. The rehabilitation process following ACLR involves a significant interaction between psychological and physical components. The psychological readiness of patients, as evaluated by the ACL‐RSI, might be impacted by their physical readiness and their capacity to resume athletic activities. Similar to the Dutch and Persian iterations of the ACL‐RSI, the Injury‐Psychological Readiness Return to Sport (I‐PRRS), a different psychological assessment scale, was employed to evaluate the accuracy of their measures. In this study, we employed physical readiness‐related scales to establish the validity of the components under investigation, thereby offering valuable insights into their relationship. Additionally, the utilization of physical readiness questionnaires can serve to guarantee that ACL‐RSI(Per) accurately assesses its intended target, which is psychological readiness for RTS, rather than solely focusing on physical preparedness [3].

According to research by Tripp et al. [40], it is demonstrated that negative emotions significantly impact athletes' performance, and the apprehension of reinjury is linked to a decreased likelihood of resuming participation in sports. In our short ACL‐RSI(Per) assessment, we observed a strong association among the six components. In our sample, the internal consistency of the overall ACL‐RSI score, as assessed by Cronbach's alpha, was 0.901. These findings align with previously reported values for various translations, indicating strong dependability of the scale in assessing the fundamental psychological dimensions. The slight discrepancies in our ICC and Cronbach's alpha values, in comparison to other studies, may be attributed to variances in sample sizes or durations of follow‐up between investigations. In summary, our findings confirm the dependability of the Persian ACL‐RSI, as indicated by the Italian version [39] of ACL‐RSI, which reported the highest alpha value of 0.99. The alpha values of our scale were much higher than those of the Arabic and Portuguese short versions, which were 0.734 and 0.78, respectively (Table 5).

The short ACL‐RSI(Per) scale shows a strong positive correlation with both the CKRS and the HSS ACL‐SS. A moderate yet significant association existed between the short ACL‐RSI(Per) scale and other variables. The investigation of the scale's translation into different languages (Table 5) has produced identical results. In this study, the correlation between the short ACL‐RSI(Per) and the Lysholm scale was found to be 0.467, which is similar to the correlations seen in the Turkish (*r* = 0.45) and Korean (*r* = 0.535) versions. The Persian version of the ACL‐RSI subscales demonstrates construct validity through its strong correlations with other scales like as CKRS and HSS ACL‐SS, which assess related variables. The slight discrepancies in correlation values observed in comparison to previous studies may be attributed to cultural or clinical disparities among the sampled populations.

Recent findings have demonstrated that the Tenger score is an effective prognostic indicator for the resumption of preoperative athletic performance. The Tenger score, similar to ACL‐RSI, proved to be a valuable tool in determining whether persons would RTS at the same level. In this study, there was a strong positive correlation (*r* = 0.414) between the ACL‐RSI(Per) and Tenger score. This connection was found to be similar to the previously published findings for simplified Chinese [8] (*r* = 0.695) and Lithuanian [32] (*r* = 0.303) versions. It is important to remark that neither of the mentioned questionnaires assessed the association between each subscale. Both the risk appraisal and confidence in performance subscales showed a comparable association with the Tenger score in this study, but the emotion subscale demonstrated a distinct correlation. As a result, it is unclear which specific aspect should be considered to have a distinct relationship, resulting in varied Pearson correlation values seen in these investigations.

Hart et al. [14] conducted a study suggesting that evaluating knee pain in conjunction with functional testing could be advantageous for those who have undergone ACLR. The results of our investigation revealed a substantial inverse connection (*r* = −0.356) between ACL‐RSI(Per) and the VAS pain scale (Table 4). Cherly et al. [29] showed that patients who had a fear of reinjury after ACLR were less likely to return to their previous level of activity before the injury. Nevertheless, we conducted an assessment of each subscale of the ACL‐RSI(Per) in connection to the VAS pain scale. Our findings indicate a noteworthy link alone between the emotion and confidence in performance subscales. The values are −0.361 and −0.397. In the recent study conducted by Kim et al. [16], it was found that risk appraisal had the highest correlation with RTS in patients following ACLR among all the subscales of ACL‐RSI.

Although the ACL‐RSI(Per) had a notable floor effect of 16.7%, its construct validity was deemed satisfactory due to its strong correlation with other pertinent questionnaires. Furthermore, its content validity was deemed favourable. This result is probably derived from data obtained from studies that included both highly skilled athletes and nonathlete patients (such as those with severe injuries) who have undergone ACLR. The occurrence of the ceiling effect was statistically significant in just 1% of the sample.

Psychological preparedness, identified as a ‘clinical criterion’ in a systematic review conducted by Turk et al. [41], involving 1432 patients across 47 papers, has a strong correlation with RTS. The fear of experiencing another rupture and the presence of a low ACL‐RSI score are significant factors that hinder the RTS process. Consequently, it is crucial to assess the psychological readiness of individuals during the prerehabilitation, rehabilitation and postoperative stages. Webster et al. proposed a shorter version of the ACL‐RSI questionnaire to eliminate redundant items from the original 12‐item version. This new version consists of only six items [42]. The redundancy in the lengthy version (Cronbach's alpha = 0.96) was resolved, and the short 6‐item tool demonstrated satisfactory consistency (Cronbach's alpha = 0.92) [42]. Using the short version of the ACL‐RSI in busy clinical environments can aid in identifying athletes who may encounter challenges in RTS following an injury.

## LIMITATIONS

There are certain limitations to this study. The psychometric validation study did not focus on RTS rates but rather used them as background context. Consequently, we were unable to assess the response of ACL‐RSI(Per). The diagnostic tool's responsiveness indicates its ability to accurately identify clinical treatment outcomes. Our experiment lacked a control group to assess the discriminatory ability of ACL‐RSI(Per). In addition, the short form was only compared to other outcome measures not directly to the original long form through correlational analysis, which would have further validated it against the established version. Future studies should directly evaluate the short form relative to the long form. The variable timeframe of follow‐up visits could have influenced the psychological readiness scores of short ACL‐RSI(Per).

## CONCLUSION

The short ACL‐RSI(Per) scale is a reliable and valid instrument for evaluating the psychological readiness to resume athletic activities following ACL surgery in Persian individuals.

## AUTHOR CONTRIBUTIONS

Peyman Mirghaderi, Nasim Eshraghi, and Seyed Mohammad Javad Mortazavi contributed to the study conception and design, wrote the draft and edited the manuscript. Mohammad Sajjadi and Reza Omid contributed to the study design, analyzed the data, data collection and wrote the first draft of the manuscript. Reza Hosseini‐Dolama and Amirreza Pashapour‐Yeganeh contributed to the study design, revised the manuscript, data collection and drew figures. Alireza Moharrami and Amir Rakhshan did the translation, was responsible for the linguistic works and edited the final version of the manuscript. Nasim Eshraghi and Payman Rahimzade answered the reviewer's comments, participated in the study design, and revised the manuscript. All authors commented on previous versions of the manuscript and revised it. All authors read and approved the final manuscript.

## CONFLICT OF INTEREST STATEMENT

The authors declare no conflict of interest.

## ETHICS STATEMENT

All procedures performed in studies involving human participants were in accordance with the ethical standards of the national research committee and with the 1964 Helsinki Declaration and its later amendments or comparable ethical standards. Tehran University of Medical Sciences' IRB approved the study. (IRB approval ID: IR.TUMS.IKHC.REC.1401.156). All patients provided written informed consent before participation.

## Supporting information

Supporting information.

Supporting information.

## Data Availability

The data that support the findings of this study are available from the Tehran University of Medical Sciences, but restrictions apply to the availability of these data, which were used under license for the current study and so are not publicly available. Data are however available from the corresponding author (Seyed Mohammad Javad Mortazavi, smjmort@yahoo.com) upon reasonable request and with permission of the Tehran University of Medical Sciences.
