# Supplementary material for: Anterior cruciate ligament—Return to sport after injury scale brief version after ACL reconstruction: Persian translation, cross‐cultural adaptation and validation
Source: J Exp Orthop. 2024 Jul 8;11(3):e12074. doi: 10.1002/jeo2.12074 (PMC11228620; doi:10.1002/jeo2.12074)
Supplement: Supplementary file 2 — Supporting information. [file JEO2-11-e12074-s001.docx]

***Short ACL- RSI Scale: Persian Translation, Cross-Cultural Adaptation, and Validation***

**پرسشنامه کوتاه ACL-RSI (رباط صلیبی قدامی – بازگشت به ورزش پس از آسیب)**

نام و نام خانوادگی بیمار: ........................................ سن: .......................... تاریخ: -- / -- / ----

**بررسی مشارکت ورزشی پس از جراحی بازسازی ACL**

دستورالعمل: لطفاً به سوالات زیر در مورد ورزش اصلی خود که قبل از آسیب انجام می دادید پاسخ دهید. برای هر سوال، بسته به آنچه به نظر شما بهتر وضعیت فعلی زانوی شما را توصیف میکند، یک عدد بین دو حد نهایی را علامت بزنید.

| **1. آیا مطمئن هستید که می توانید در همان سطح قبلی خود در ورزش شرکت کنید؟** | | | | | | | | | | | | |
| --- | --- | --- | --- | --- | --- | --- | --- | --- | --- | --- | --- | --- |
| کاملاً مطمئنم | 100 | 90 | 80 | 70 | 60 | 50 | 40 | 30 | 20 | 10 | 0 | اصلاً مطمئن نیستم |
|  | O | O | O | O | O | O | O | O | O | O | O |  |

| **2. آیا فکر می کنید که ممکن است با انجام ورزش قبلی خود، دوباره به زانوی‏تان آسیب وارد کنید؟** | | | | | | | | | | | | |
| --- | --- | --- | --- | --- | --- | --- | --- | --- | --- | --- | --- | --- |
| اصلاً احتمال ندارد | 100 | 90 | 80 | 70 | 60 | 50 | 40 | 30 | 20 | 10 | 0 | خیلی احتمال دارد |
|  | O | O | O | O | O | O | O | O | O | O | O |  |

| **3. آیا در مورد انجام ورزش قبلی خود حس نگرانی دارید؟** | | | | | | | | | | | | |
| --- | --- | --- | --- | --- | --- | --- | --- | --- | --- | --- | --- | --- |
| اصلا نگران نیستم | 100 | 90 | 80 | 70 | 60 | 50 | 40 | 30 | 20 | 10 | 0 | خیلی نگران می‏شوم |
|  | O | O | O | O | O | O | O | O | O | O | O |  |

| **4. آیا مطمئن هستید که می توانید ورزش قبلی خود را بدون نگرانی از زانوی خود انجام دهید؟** | | | | | | | | | | | | |
| --- | --- | --- | --- | --- | --- | --- | --- | --- | --- | --- | --- | --- |
| کاملاً مطمئنم | 100 | 90 | 80 | 70 | 60 | 50 | 40 | 30 | 20 | 10 | 0 | اصلاً مطمئن نیستم |
|  | O | O | O | O | O | O | O | O | O | O | O |  |

| **5. آیا برای شما مایوس کننده است که باید هنگام ورزش مراقب زانوی خود باشید؟** | | | | | | | | | | | | |
| --- | --- | --- | --- | --- | --- | --- | --- | --- | --- | --- | --- | --- |
| اصلا مایوس کننده نیست | 100 | 90 | 80 | 70 | 60 | 50 | 40 | 30 | 20 | 10 | 0 | بسیار مایوس کننده است |
|  | O | O | O | O | O | O | O | O | O | O | O |  |

| **6. آیا از آسیب رسیدن دوباره به زانوی‏تان با انجام ورزش قبلی خود می ترسید؟** | | | | | | | | | | | | |
| --- | --- | --- | --- | --- | --- | --- | --- | --- | --- | --- | --- | --- |
| اصلا ترس ندارم | 100 | 90 | 80 | 70 | 60 | 50 | 40 | 30 | 20 | 10 | 0 | بسیار می‏ترسم |
|  | O | O | O | O | O | O | O | O | O | O | O |  |

- **نمره ACL-RSI (6 / نمره کل) = ____ %**

احساسات Emotions (سوالات 3، 5، 6) = ____ %

اطمینان به عملکرد Confidence in Performance (سوالات 1، 4) = ____ %

ارزیابی خطر Risk Appraisal (سوال 2)= ____ %
